# Feasibility of implementing rapid oral fluid HIV testing in an urban University Dental Clinic: a qualitative study

**DOI:** 10.1186/1472-6831-12-11

**Published:** 2012-05-09

**Authors:** M Katherine Hutchinson, Nancy VanDevanter, Joan Phelan, Daniel Malamud, Anthony Vernillo, Joan Combellick, Donna Shelley

**Affiliations:** 1 Colloege of Nursing, New York University, 726 Broadway, New York, NY, 10003, USA; 2 College of Dentistry, New York University, 345 E. 24th St., New York, NY, 10010, USA

**Keywords:** HIV testing, Barriers, Dental care

## Abstract

**Background:**

More than 1 million individuals in the U.S. are infected with HIV; approximately 20% of whom do not know they are infected. Early diagnosis of HIV infection results in earlier access to treatment and reductions in HIV transmission. In 2006, the CDC recommended that health care providers offer routine HIV screening to all adolescent and adult patients, regardless of community seroprevalence or patient lifestyle. Dental providers are uniquely positioned to implement these recommendations using rapid oral fluid HIV screening technology. However, thus far, uptake into dental practice has been very limited.

**Methods:**

The study utilized a qualitative descriptive approach with convenience samples of dental faculty and students. Six in-depth one-on-one interviews were conducted with dental faculty and three focus groups were conducted with fifteen dental students.

**Results:**

Results were fairly consistent and indicated relatively high levels of acceptability. Barriers and facilitators of oral fluid HIV screening were identified in four primary areas: scope of practice/practice enhancement, skills/knowledge/training, patient service/patient reactions and logistical issues.

**Conclusions:**

Oral fluid HIV screening was described as having benefits for patients, dental practitioners and the public good. Many of the barriers to implementation that were identified in the study could be addressed through training and interdisciplinary collaborations.

## Background

More than 1 million individuals in the U.S. are infected with HIV [1]; approximately 20% do not know they are infected [2]. As a result, late diagnosis of HIV infection is common; 30–40% of individuals who are newly diagnosed with HIV infection have immune suppression when first diagnosed [3-6], and many develop full-blown AIDS within one year [4,6]. Earlier diagnosis and access to care and treatment reduces transmission [4,7-10]. Early treatment with highly effective antiretroviral therapy (HAART) leads to reductions in viral load that have been associated with reduced infectivity and likelihood of HIV transmission to others [9,11]. In 2006, the CDC issued new recommendations that all health care providers offer routine HIV screening to individuals, ages 13 to 64, regardless of community seroprevalence, patient lifestyle, or perceived risk [12]. The type of widespread testing recommended could lead to the diagnosis of more than 56,000 new HIV cases per year [13]. The revised CDC recommendations represent a dramatic shift in policy [8,14]. Health care providers have been slow to implement routine HIV screening [15]; these delays may be due, in part, to perceived barriers on the part of providers [15].

Dental practice sites present unique opportunities for implementing the CDC’s recommendations for routine HIV testing [16]. Pollack, Metsch and Abel analyzed National Health Interview Survey data and found that 3.6 million persons at significant risk for HIV had not been tested; 75% had seen a dentist in the past two years [16]. The authors concluded that HIV testing in the dental setting had great potential for reaching high risk populations [16]. A recent demonstration project found significant success implementing HIV testing in the dental setting. Harlem Hospital Center screened more than 3500 dental patients over a 22-month period using rapid OraSure testing of finger-stick blood [17]. The vast majority of patients who were approached (97.6%) agreed to be tested. Nineteen patients who were previously undiagnosed screened HIV+, an incidence rate of 0.55%. Of these, 15 completed follow-up confirmatory testing by Western Blot. All 15 were confirmed positive and were linked to care. Of these, 40% met the diagnostic criteria for AIDS. (p. 782) [17]. Although this study highlighted the potential of dental practice sites to provide HIV testing to patient populations, it must be noted that HIV testing and counseling services in this study were provided by a trained counselor, not by a dentist [17].

### Dental scope of practice and training

Although the traditional role of dentists may have been viewed as limited to the oral cavity, a broader and more integrated vision of dentists as healthcare partners with physicians and other health care providers has been endorsed by some dental leaders [18,19]. Many dentists consider health screenings important and incorporate them into their practices [20]. Given the recent explosion in technology that allows oral fluid testing for a wide variety of health conditions including HIV [21,22], this aspect of dental practice is likely to continue to expand. In addition, in many ways dentists are already on the diagnostic frontline of HIV/AIDS, as oral manifestations of immune suppression are seen in dental practice [23-25].

In a 2001 survey of U.S. dental schools (85% response rate), all programs reported curricular content on HIV behavioral risk, oral manifestations of HIV, and the importance of a comprehensive medical history [18]. However, nearly 40% reported their dental curricula provided students with little or no training in how to refer patients for HIV counseling and testing. Further, more than 60% of dental schools reported that their dental clinics never screened for HIV and 22% rarely screened. While more than 30% of dental program respondents indicated they would consider testing in their dental clinics, many expressed concerns about the ability of dentists to conduct such testing because they lacked training in HIV testing and counseling [18].

### Rapid oral HIV diagnostic technology

The availability of rapid diagnostic test kits that are capable of detecting HIV-1 and HIV-2 antibodies in oral fluid has greatly facilitated the acceptability and potential for widespread HIV testing in dental sites and elsewhere [8,26]. Malamud described saliva HIV testing as “a technology whose time has come” (p. 9) [21]. Rapid oral saliva screening tests for HIV antibodies have high sensitivity and specificity [4,21], although positive results are considered preliminary and require confirmatory Western Blot (or similar) testing [21]. Rapid oral test kits are convenient, easy to use and provide results in approximately 20 minutes [21]. Oral fluid testing is safer for providers in that it eliminates the risk for needlestick injury and transmission of HIV virus from oral fluid is unlikely [21]. Following advances in oral HIV testing technology and the CDC recommendations, calls to integrate HIV testing in dental practice have increased among leaders in dentistry [4,18,19,26].

Consistent with the recommendations of these dental leaders, the CDC and the National HIV/AIDS strategy, the purpose of this interdisciplinary study was to examine the feasibility of implementing rapid oral HIV screening in a large, university dental admissions clinic in New York City. Specifically, we sought to understand providers’ attitudes, beliefs and perceived barriers to screening in order to address these factors in an implementation plan.

## Methods

The study utilized a qualitative descriptive approach [27], in which data were collected through in-depth one-on-one interviews with dental faculty and through focus groups with dental students. The qualitative approach was appropriate in order to gain insight into and explore dental faculty and students’ perceptions and beliefs regarding the feasibility and acceptability of incorporating saliva HIV testing into dental clinic practice.

### Setting and sample inclusion criteria

The study took place at the New York University College of Dentistry (NYUCD) in Manhattan. NYUCD is the single largest “safety net” provider of dental care in New York State, and provides comprehensive oral health care to 124,000 patients (300,000 visits) each year. The population served is 60% African American and Hispanic; 56% are Medicaid insured [28,29]. Haber and associates surveyed new dental patients at NYUCD (2,580 surveys; 946 responses; 36.7% response rate) and found that 33% did not have a primary care provider and 27% reported having no medical insurance [28]. Many patients come from the highest HIV seroprevalence zip codes within New York City’s five boroughs. All new patients who present at the NYUCD for care are first seen in the admissions clinic, the setting for the current study.

Routine oral fluid HIV screening is not currently offered in the admissions dental clinic or other clinics within the NYUCD by dental faculty or students. Some of the dental faculty may have previous experience with oral fluid HIV screening and/or may be conducting such testing in other clinical practice settings. A pilot study of oral fluid HIV screening was conducted in the NYUCD in 2008–2009; in this study, patients were much more likely to agree to be tested when it was offered by a dentist compared to when patients had to request screening [unpublished data]. However, at the time the pilot study was conducted, only a few of the dental faculty participated. The current study sought to build upon the findings of the earlier pilot study in order to plan for widespread routine HIV screening in the NYUCD admissions clinic.

The study included a purposeful sample of dental faculty who provide care in the NYUCD admissions clinic and a convenience sample of third and fourth year dental students who have completed clinical rotations in the admissions clinic. Data collection continued until data saturation occurred and no new information was obtained [30,31]. Inclusion criteria included: a) 18 years of age or older (which all dental faculty and students are); b) able and willing to agree to participate; c) able to speak and understand English; and either d) NYUCD 3^rd^ or 4^th^ year dental student or e) NYUCD faculty dentist who works in the admissions clinic at least 4 hours/week. Admissions clinic faculty who were radiologists and only worked in the radiology area of the admissions clinic were excluded from participation. The final sample included six dental faculty and 15 dental students.

### Data collection procedures

Written consent forms were provided to all potential study participants although signed consent forms were not collected as these would have provided the only participant identifiers. Consent was determined by willingness to participate after reading the written informed consent form. All study participants were reassured verbally and in writing that responses would be kept strictly confidential and would not be linked to demographic data or descriptors that could lead to deductive disclosure. All procedures were reviewed and approved by the Institutional Review Board of the New York University School of Medicine prior to data collection.

#### Dental faculty

A purposeful sample was recruited from among the 17 dental faculty who provide direct care and supervise students in the dental admissions clinic. Participants were selected from a list of 23 admissions clinic faculty; the list included names, email addresses, gender, age, number of hours per week worked, specialty and whether or not the individual practiced outside of the admissions clinic. Six of the 23 faculty were radiologists and were excluded. The goal for sampling was to obtain demographic representation and variation in the phenomena of interest [32]. Recruitment began by emailing contact letters and consent forms to four potential faculty participants (2 men and 2 women) inviting them to participate in face-to-face interviews. Interviews were scheduled at the participant’s convenience in his/her office or in a quiet private location of his/her choosing. If a selected dental faculty member did not respond after two contact attempts, then another who was similar in age, sex, years of experience, etc. was selected in his/her place. In total, nine admissions clinic dental faculty were contacted. Six one-on-one interviews were completed; each interview lasted approximately 45–90 minutes. Dental faculty did not receive reimbursements or incentives for participating.

Interviews were conducted by one of the investigators (MKH) who has extensive experience with qualitative interviewing and focus group research. In addition, she was not known to the dental faculty or student participants. Semi-structured interview guides with open-ended questions and probes were used to assess dental faculty members’ attitudes, beliefs, perceived barriers, intentions and experiences related to HIV saliva testing in the admissions dental clinic and dental practices in general. Questions progressed from the general to the more specific.

#### Dental students

A convenience sample of 15 dental students was obtained from among the approximately 700 third and fourth year dental students at NYUCD. The IT director at NYUCD initially generated a random sample of 80 names from the roster of approximately 350 fourth year dental students. A contact letter and consent form was sent to each of these students explaining the study and inviting his/her participation. Interested students contacted the research assistant and 5 students were scheduled to participate in the first focus group. Because response rates were low, purposeful sampling was not feasible. Participation was subsequently opened up to all third and fourth year students. Interested students contacted the research assistant and were scheduled into focus groups.

Dental students were provided with food and received $10 cafeteria vouchers for their participation. Focus groups were held in a private conference room and facilitated by one of two investigators (MKH or NVD), both of whom are experienced focus group facilitators. A focus group guide was developed based upon the Dentist Interview Guide and informed by the results from the first few dental faculty interviews. Fifteen dental students participated in 3 focus groups.

#### Data collection and analysis

Data collection and analysis were conducted as iterative processes. The final faculty interview and focus group established informational redundancy when no new information was obtained [30-32].

## Results

Audio-taped interview and focus group data were transcribed verbatim and edited to remove any identifiers. Transcripts were read thoroughly multiple times and coded independently by two of the investigators (MKH and NVD) with extensive experience in qualitative data analysis. Initially, primary codes that related to the analytic foci of the study were developed, followed by sub-codes that specified specific dimensions of primary codes. In addition, text was coded using categories developed from the data themselves [30,31]. Nonverbal behaviours and other observations were recorded by the research assistant [33]. The two data coders met and, along with the research assistant, compared coding and resolved discrepancies.

Dental faculty and students identified positive factors that would facilitate HIV screening in the admissions dental clinic and negative factors (barriers) that would make implementation of HIV screening more difficult. In general, results were fairly consistent among dentists and dental students and are presented in four primary areas: scope of practice/practice enhancement, skills/knowledge/training, patient service/patient reactions and logistical issues.

### Scope of practice/practice enhancement

Nearly all of the dental students and most of the dental faculty described HIV screening as being within the dental profession’s scope of practice. Typical responses included: “We are health professionals”; “This is oral diagnostics. I mean if we’re not going to do it, who the heck, who else is?”; “People come to us more often than they see their primary care physicians”; “We do oral cancer screenings, so why not do [oral] HIV screening too?”; and “For us, this is a great tool. It’s a simple thing, just take some saliva and do the test.” Most participants did not see this as a conflict with medicine. As one participant stated, “And I don’t think that’s going to be taking away from physicians. We’re going to be helping. We’re going to be sending patients to them or nurse practitioners. We’re not going to be taking away from the practice. If anything, we’re going to augment it and help them help us.” Some referred to dental HIV screening as “breaking the mold” for dentistry. “We start it, and then people go [to a medical health care provider] and are like, ‘Oh, well, my dentist already did that [HIV testing].’ Well, now, that’s kind of breaking the mold and that’s a great thing.”

In contrast, one or two comments were made questioning whether oral HIV screening fell within the dental scope of practice. “Is the dentist really the one who has to do it or is [it] really a medical provider?” A number of dentists and dental students referred to “generational effects” in attitudes toward HIV screening. These generational effects seemed to be unrelated to age or generation, per se, and more akin to more traditional or conservative views of dental practice. For example, “If there’s any resistance, it’s probably going to be the older guard who have come from a generation where this wasn’t really part of practice, and only because they’re not comfortable with it, they’ve never had to deal with it.” “You would definitely get a wide spectrum of people. Like, people who would say, ‘That’s not my job.’” On a similar note, “Why are we doing this? This should be someone else’s responsibility.” “I know some faculty and some students who are like, you know, ‘I just want to drill and fill. . . . why do I have to learn all this other stuff?’” Those who were in favour of incorporating HIV screening into practice took issue with the notion that scope of practice was a legitimate barrier or argument against it. One shared a recent experience at a professional meeting where dentists were being trained to give Botox injections. The respondent concluded with “It rankles me a bit” and “It’s amazing how quickly those barriers melt when you see an opportunity for something different.”

#### Practice enhancement

Many dental students and some dental faculty expressed the view that HIV screening and oral diagnostics, more broadly, were the wave of the future. For example: “Saliva testing is a paradigm change for dentistry—we can only go forward.” “I think this is a very exciting time for us.” “[Oral diagnostic testing] has basically changed the healthcare paradigm of dentistry and it’s changed irreversibly and we can only go ahead.““We’re going to see more and more of these things [oral fluid diagnostic tests] coming up.” “This is really also part of the more general thing that I see coming down the pipe which is oral diagnostics in dentistry exploding.” Many went on to describe oral fluid HIV screening as a form of practice enhancement and a means of expanding the profession’s scope of practice.“This is an opportunity to branch out…..and that’s smart” “This has got to change the paradigm of dentistry forever and it’s got to make a much more exciting opportunity for us as dentists to interface with the public and refer them to physicians and nurse practitioners.” “It takes you a few minutes. But when a patient sees when a dentist is really being thorough with them, they walk away saying ‘hmmm’. That’s how you sell your practice right there.”

Dental students reported that training in HIV screening and other oral fluid diagnostics would be “cutting edge” and helpful in their future practices. Nearly all of the dental students expressed the desire to be trained these techniques: “Students need to be trained; we are the next generation.” Another offered, “I think it would be a good experience for the students to get their foot in the door with oral diagnostics because it is going to become a huge field at some point in our career, in the next ten, fifteen years, probably sooner. Getting in at the ground floor would really be, probably, a priceless experience.” Faculty concurred: “…We continue to train students. That doesn’t necessarily mean a 1:1 correspondence, [not] every student who is trained to do HIV testing as a dental student is going to be doing it as a practitioner, but what it is going to do is take them beyond.”

Beyond scope of practice, respondents described issues of skills, knowledge and training that would promote or inhibit the implementation of HIV screening in dental practice. Both strengths and needs for further training were identified.

### Skills/knowledge/training

As was described above, most participants reported that oral rapid HIV screening fell within dentistry’s scope of practice. Students acknowledged that they were learning about oral diagnostic techniques. Some said, “we get enough on a scientific point of view . . . “, while others reported needing and wanting more: “We never had a course teaching us how to test the patient.”; “Why not have a skill set?” “Just to keep everyone’s options open. It’s a great thing to learn as long as the testing is accurate.” Nearly all of the participants, dentists and students alike, made direct or indirect references to the need for training—training in testing procedures, how to communicate with patients about HIV testing and results, and referral procedures. Some mentioned the need for further training in the use of the oral fluid testing technology (e.g., OraSure OraQuick Advantage): “Mechanics of the test. The AETC [AIDS Education Training Center] website—you can get [training] on that.” Some students felt that HIV testing should be taught in the classroom, while others believed it should be taught in the clinic. “Whatever relates to clinic, I think, should be taught in the clinic.”

Many seemed undaunted by the prospect of testing, provided they were given training. “I don’t think the technical part of us doing the test is an issue.” Another offered: “You don’t have to have 3 PhDs in astrophysics to do this.” Students and dentists alike mentioned the need for dental students to be trained in screening procedures and to have opportunities to practice and maintain skills. “If they don’t do it [HIV testing], they will forget.”

#### Discomfort with HIV-related communication

The actual testing procedures seemed less worrisome to dental providers and students than patient counselling and communication components. For example, “We do know about the testing and stuff, but I think it’s more about from the emotional standpoint.” Some dental faculty and many dental students expressed discomfort with the “seriousness” of communicating with patients about HIV screening and findings. “. . . I think. . . all the dentists. . . we’re very good talking about teeth and even oral health. . . but when you get to certain questions it’s still, I think, it’s a barrier.” Discussing HIV screening results with patients was described as being even more daunting: “Testing is the least of the problem; what happens next is.” “What’s more important . . . is the ability to counsel and deal with people that do come back positive. . .” “Dentists . . . are not prepared to deliver really bad news.” Dental students, in particular, expressed the need for training and the desire to develop expertise in this type of patient communication: “I think a screen that can be done orally does fall within the dentist’s scope . . . but I think what’s more important than that is the ability to counsel and deal with people who do come back positive.” “As far as dealing with that, I agree . . . it’s not like we’re prepared for [giving results]. I don’t even have the capacity to deal with the emotional involvement that goes with announcing that.”

Both dental faculty and students discussed the need and potential benefits of training. For example, “Being uncomfortable is understandable but [we] can be trained to counsel patients.” Several training strategies were discussed by participants, including the use of written protocols, scripts and practice role plays to help develop skills and comfort with HIV-related patient communication. “I believe students need to be given the standard protocol for how it must be done.” “. . . there should be some kind of role playing, like, of delivering the news and stuff like that. That’d be really key in helping someone get past the barrier.” This dental student offered the analogy of practice drilling and various dental procedures before doing them in the clinic with patients. “I’m going to tell you, not [everyone] will do it right.” Regarding role modelling and role plays, one offered: “There has to be some kind of support system and training system.” “Sometimes you have to hear somebody else. It’s the same thing with the students.” Some referred to the need for role modelling by faculty: “The student sees how a dentist interacts with the patients. It’s part of the student’s training as much as, you know, you’re preparing a crown for a tooth. I think all of that becomes a way of learning how to interact with patients and learning how to conduct yourself in practice.” In response to questions about scripting, one faculty stated, “Yes we’re very good following scripts and instructions.”

Participants’ discomfort with giving HIV results led some to suggest that dental patients should get their results elsewhere at a later time. Quotes included: “. . . there might have to be HIV clinics to go to for results” and “I think that we can perform the test and send the results to the lab or whatever, and then have a universal centre where patients could get results.” “I think that would be easier on the practitioners, dentists and the patients as well . . they might feel more comfortable in that type of setting.” “Maybe we could mail results.”

### Patient service/patient reactions

Both faculty and students described “benefitting the public good” as the primary reason to undertake HIV screening: “You’re going to be contributing to the public good”; “I think in that setting [admissions clinic] we can screen a lot of people and explain to them the merits of doing it.”; “People know about HIV infection today. We’re 25–30 years down the road from the [start of the] epidemic, so people have a pretty good idea of what causes it. . . .I don’t think the public is as unknowing about it as we were before.““The New York City public is very savvy about HIV.” Nearly all students and most dental faculty believed that patients would view HIV screening as a benefit. “As far as my experience, little experience so far with my patients, most of the times, like 99% of the time, they’re very appreciative when we discuss their systemic health or bring up things like their blood pressure, anything that has to do with their health and well-being and we cover all the bases. I always get very positive feedback from my patients when we’re thorough like that. I think it [HIV screening] would be very appreciated by our patients.” “I think they will love it.” “I’ve never had resistance towards any of the testing that we offer to them.” Participants were also positive about New York State laws regarding “opt-out” procedures for HIV testing; the majority felt that “opt-out” would make implementation in dental practices more feasible. “The idea that you don’t have to sign a consent form, I think, should make it a lot easier.” “. . . If you want it—fine; if you don’t, tell me and I won’t test you.”

A few of the dental faculty expressed concerns that patients would react negatively to HIV screening and/or offers of HIV screening. Comments included, “I don’t know if the patient sees us [as] the provider for that.” Another offered: “Patients don’t want to know; there is still stigma.” One dentist felt that offering HIV screening would be a deterrent to care; “If you do it for everyone, patients will not want to come to the clinic.” “They might not want us to know.” Two faculty members believed that HIV screening should be limited to “high risk” patients. “Patients won’t see the advantage to being screened when they have to get confirmatory testing and they will ‘freak out’ [if they screen positive].” The contrasting view was expressed as: “When people come into a dental operatory we check them for carries. Doesn’t make any difference whether they’re men, women, 7 feet tall, 3 feet high, orange, green, pink, whatever. It doesn’t make any difference, we look for these things. So if we’re going to fold HIV infection into routine dental care, it should make no difference. We should test everybody.”

### Logistical issues

A number of logistical issues were identified that could affect the feasibility of implementing HIV screening in the dental admissions clinic. These included issues and limitations related to time, resources, cost, space, patient confidentiality and referrals. Despite these limitations, the admissions clinic was identified as the best site for HIV screening within an academic dental setting. The “fit” was attributed to the clinic’s focus on assessment and diagnosis. “I think it’s just easier for us to do it [in the admissions clinic] because that’s where we really sit down and talk to people about their health.” “It fits.” Although participants identified several logistical issues that would need to be addressed in the admissions clinic, many felt these issues would be even more problematic in private practice. “Might work in a dental clinic environment but not necessarily in the [private] dental office.”

#### Time and personnel constraints

One of the most commonly cited logistical challenges was time constraints. For example, “How much more [are] the dentists here going to expand the examination?” “It’s like the idea . . . . is to put more responsibility on the dentist. . . in the small time frame we have. When the patient comes with a chief complaint, it’s more related with the teeth. . . .we have to talk a little bit more and start with different kind of testing.” Concerns were also expressed about a potential loss of income if time was taken for HIV screening and referral. One mentioned that this issue could be more intense for new graduates. “In the beginning they [dentists] work for somebody else. [They] come out with debt from dental school. . . They still need to live their life and pay all the loans. I cannot blame them for that.”

Others disagreed that time constraints were significant barriers to HIV screening in the admissions clinic: “Yeah because you have a lot of [time] gaps between seeing the patient, taking the X-rays, taking the medical history.” Even if private practice settings, “There is no reason that a dental practice has to come to a screeching halt to do HIV testing, even if it is indeed, understandably, a very productive and busy practice. ““You can train a hygienist to take this test on a patient.” “. . . hygienists could do it in private practice, and only if the test result comes out positive, then you know, dentists [intervene] at that point. Deliver the news.”

There was consensus that some providers might be better suited than others to perform HIV screening and give results. Several suggested that, at least initially, HIV screening in the admission dental clinic should be done by a few specially trained persons. “It probably would be better to have a few people who are actual counsellors because we [students] keep moving . . . and then we graduate. . . having a couple counsellors, maybe even the nursing people can do that. I don’t know. They can do it for a longer time and a better job because they develop their skills.” Other quotes included: “I don’t think it should ever be all of the faculty. It should be specially trained people.” “I don’t think some of them [faculty] would want to do it.”

#### Costs and reimbursement

Beyond time constraints, participants expressed mixed opinions regarding the costs and reimbursements for HIV screening and services. Some believed dentists could bill for these services: “You can bill for the pre-counselling. You can bill for the test.” “Also they can bill for this. You can put this under a medical code and insurance will pick it up. You have to look at the state laws. You have to look at what insurance covers what.” Others believed that dentists could not bill for these services: “You have a lot of things in dentistry that are coming out . . . and the evidence is supporting that technique but you cannot do it because . . . third party payers; it’s like you will not get paid to do that.”

#### Space, patient confidentiality and referrals

The most serious concerns and barriers to HIV screening expressed by dentists and students alike related to issues of patient confidentiality—e.g., needing a private area to give results, being able to protect the patient’s privacy, and being able to refer to patient for immediate follow-up testing and care. For example, “[where to give results to patients] That, I think, is a critical piece.” Other quotes included: “I really do struggle with the confidentiality.” “I think the thing you’d want to make sure of in a clinic that busy with people buzzing all around is that you’re keeping that information confidential. . . .that the person in the next cubicle isn’t overhearing. That’s the only concern I have.” “. . .we have to be careful and we have to teach our students what we’re going to say in front of whom.. . . . these cubicles, you can hear things. You’ve got to be subtle.” “I just think the way the [clinic] is set up, it’s not private at all. So patients don’t want to discuss that in front of everyone. . . . The cubicles are right next to every other patient. It would be difficult to do it like that.” Other students felt that the admissions clinic was “a good place [for HIV screening].”

There was consensus among dentists and dental students that there was a need for standardized procedures for offering testing. “I think that one thing that could make it easier . . . we group it with other tests, like, ‘Oh we screen for oral cancer and [HIV] here, are you interested in doing that?’” Standardized procedures were seen as very important for giving results, whether the HIV screening results were positive or negative: “. . . .like, everyone claim your results over here.” “[You need] a designated location. That way, everyone’s doing the same kind of thing.” “If every person getting screened goes into an office or something afterwards, to like go through the counselling part of it; that would be more appropriate.” Faculty and students discussed the need to revise the medical history intake form as the current form only asks if the patient has HIV. “In the paperwork, . . .you ask questions about smoking, and the next line on the same paper is asking about alcohol. I think the next line should be, ‘Have you ever been tested for HIV?’” ‘Have you ever been tested? Would you like to? We offer . . .”

Referrals were another serious concern: “I would say you have to be trained in that aspect [referral].” “If you get a [positive] result you have to be in a position to refer the patient properly.” “How to follow through with them and where the next step is, is really what’s probably most important.” When asked where they currently refer patients with health problems, students mentioned, “We refer them to the nurse practitioner.” Some felt that referrals and timely linkage to care might be easier in urban areas. For example, “When you’re in New York City, we’ve got healthcare professionals all over the place. We have our nurse practitioners here. We’ve got physicians everywhere else. There’s no problem getting a patient with a positive screening result to a healthcare professional who can render a diagnosis.”

## Discussion

### Perceived benefits of oral fluid HIV screening in the dental clinic

The majority of dental providers, both faculty and students, described rapid oral fluid screening for HIV as consistent with the professional scope of practice and, in many instances, the cutting edge and the future of dentistry. Oral fluid diagnostic testing was seen as an emerging field that would be expanding in the near future, and an area that held great promise for dentistry. The link between oral fluids and the dental profession was seen as obvious. Great enthusiasm was expressed for how oral fluid diagnostics, including HIV testing, would change or enhance the field of dentistry in the future; the word “exciting” was often used to describe the emergence of this new practice area. Oral fluid HIV testing, and oral fluid diagnostics more broadly, were also described as a means to better integrate dentistry with other health professions.

HIV screening in dental clinics was also described as contributing to the public good. Most participants agreed that offering testing would provide a valuable service to the NYUCD dental patients and also benefit public health. A few participants expressed concerns that offering HIV testing could potentially harm the dental clinic practice because patients might want to avoid being tested.

### Perceived barriers

A number of barriers to implementing HIV screening in the academic dental admissions clinic were also identified. The most commonly cited barriers included: concerns over negative patient reactions; logistical issues related to time, cost, space and patient privacy issues; and discomfort related to communicating about HIV and test results with patients. Dentists’ and dental students’ perceived barriers were consistent with those identified in two earlier studies [18,34]. Identifying perceived and developing strategies to overcome them can reduce resistance and facilitate behaviour change [35].

#### Potential for negative patient reactions

Concerns regarding the possibility of negative patient reactions to being offered HIV screening were mentioned by a few participants. However, recent studies indicate high levels of patient acceptance. Nearly 75% of patients at a Kansas City, Missouri dental clinic reported that they would be willing to take a free rapid HIV screening test [3]. Rates were even higher (91%) among Hispanics and may reflect a lack of other sources of routine care. Even higher rates of patient acceptance (97%) were noted in a Harlem dental clinic, despite the fact that testing involved rapid testing of whole blood via fingerstick [17]. Patient data from the current study site (midtown New York City) were consistent with the findings from Harlem [17], in that the vast majority of adult patients who were interviewed reported that they would be willing to be tested for HIV [36]. Differences between patients’ attitudes in New York City and Missouri may reflect geographical differences in attitudes and/or differences in HIV seroprevalence rates. Such differences in patient acceptability need to be addressed in local implementations.

#### Logistical constraints

Study participants identified several logistical constraints that would be important to consider prior to implementing HIV screening in the admissions dental clinic. Two of the most important were limitations in the physical space and layout of the clinic and the resultant lack of patient privacy. These were seen as critically important barriers that would have to be effectively addressed in any implementation plan. All of the participants either directly identified patient privacy as a key barrier or concurred when other focus group members mentioned it. While patient privacy is always an important consideration in any practice setting, concerns were heightened because of the numbers of patients being seen at the same time and the lack of full walls and doors on the operatories. Patients are seen in cubicles that are separated by partial walls. Discussing HIV risk and, even more importantly, HIV screening results with patients without adequate privacy was seen as a serious potential problem.

#### Discomfort communicating about HIV

In addition to patient reactions and patient privacy, dentists and dental students were very concerned about being able to effectively communicate with patients regarding HIV testing, risks and results. While some providers believed these skills could be developed, others expressed serious concerns about the emotional toll associated with conveying positive HIV screening results to patients.

### Limitations

The study findings should be viewed in light of the study limitations. The small sample size (21) was consistent with the qualitative method and was not a study limitation per se, as data saturation or informational redundancy was reached [30-32]. However, although the sample of dentists was diverse and purposefully selected, the sample of dental students was a small convenience sample. Those who were most opposed to HIV screening in dental clinics or uncomfortable discussing HIV may have been less likely to agree to participate. As such, their voices would not have been heard. Further, although generalizability per se is not the aim of qualitative research, the barriers to implementing HIV screening may be very different in private dental practice settings.

## Conclusions

Dentistry has the ability to play a vital role in implementing widespread HIV testing [16,26]. The current study identified multiple benefits of oral fluid HIV testing in academic dental clinics; benefits were identified for patients as well as for dental professionals. In addition, a number of barriers were identified that could hamper effective implementation. The study results highlighted the importance of conducting formative assessments to identify such barriers and develop effective strategies to address them prior to implementing HIV testing in the dental clinic setting. For example, reconfiguring space within the clinic may be necessary in order to provide a private, quiet area where HIV screening test results can be discussed with patients.

Some of the other barriers identified in the current study (e.g., discomfort with HIV-related patient communication) can be addressed through training and/or interdisciplinary collaborations. For example, communication training, the use of scripts, role plays and role modelling may all contribute to dental providers’ comfort and competence in communicating with patients about HIV risk and test results. In addition, interdisciplinary collaborations between dentistry and nursing, medicine or other health care professions may present useful models to address many of the barriers identified in the current study, including time constraints, communicating results to patients, needs for confirmatory testing and referrals, and billing for services. These types of multi-disciplinary approaches are consistent with recommendations from the Institute of Medicine (IOM) and others to re-engineer care processes, broker knowledge and workforce skills, foster interdisciplinary team-building and reinforce care coordination [28,29,37], and American Dental Education Association (ADEA) recommendations that dentistry develop interdisciplinary models of care that integrate other primary care providers as team members [38]. Nurse practitioners may be ideal potential collaborative partners for dental sites seeking to implement HIV screening into practice. Nurse practitioners (NPs) are cost-effective primary care providers [39] who practice independently or collaboratively with physicians; their services are reimbursable by Medicaid and Medicare [40].

## Competing interests

The authors report no competing interests regarding this paper.

## Authors’ contributions

All authors contributed equally to the design and execution of the study. All authors read and approved the final manuscript.

## Pre-publication history

The pre-publication history for this paper can be accessed here:

http://www.biomedcentral.com/1472-6831/12/11/prepub
